# Insights into the Phytochemical and Multifunctional Biological Profile of Spices from the Genus *Piper*

**DOI:** 10.3390/antiox10101642

**Published:** 2021-10-19

**Authors:** Simon Vlad Luca, Katarzyna Gaweł-Bęben, Marcelina Strzępek-Gomółka, Karolina Czech, Adriana Trifan, Gokhan Zengin, Izabela Korona-Glowniak, Mirjana Minceva, Jürg Gertsch, Krystyna Skalicka-Woźniak

**Affiliations:** 1 Biothermodynamics, TUM School of Life Sciences, Technical University of Munich, 85354 Freising, Germany; vlad.luca@tum.de (S.V.L.); mirjana.minceva@tum.de (M.M.); 2 Department of Cosmetology, University of Information Technology and Management in Rzeszów, 35-225 Rzeszów, Poland; mstrzepek@wsiz.edu.pl (M.S.-G.); kczech@wsiz.edu.pl (K.C.); 3 Department of Pharmacognosy, Grigore T. Popa University of Medicine and Pharmacy Iasi, 700115 Iasi, Romania; 4 Physiology and Biochemistry Research Laboratory, Department of Biology, Science Faculty, Selcuk University, Konya 42130, Turkey; gokhanzengin@selcuk.edu.tr; 5 Department of Pharmaceutical Microbiology, Faculty of Pharmacy, Medical University of Lublin, Chodzki Street 1, 20-093 Lublin, Poland; iza.glowniak@umlub.pl; 6 Institute of Biochemistry and Molecular Medicine, University of Bern, CH-3012 Bern, Switzerland; juerg.gertsch@ibmm.unibe.ch; 7 Independent Laboratory of Natural Products Chemistry, Medical University of Lublin, 20-093 Lublin, Poland; kskalicka@pharmacognosy.org

**Keywords:** *Piper*, piperamides, antimicrobial, anti-tyrosinase, anti-melanogenic, natural bioactive agents

## Abstract

*Piper* spices represent an inexhaustible reservoir of bioactive compounds that may act as drug leads in natural product research. The aim of this study was to investigate a series of methanolic fruit extracts obtained from *P. nigrum* (black, green, white and red), *P. longum* and *P. retrofractum* in comparative phytochemical and multi-directional biological (antimicrobial, antioxidant, anti-enzymatic and anti-melanogenic) assays. The metabolite profiling revealed the presence of 17 piperamides, with a total content of 247.75–591.42 mg piperine equivalents/g. Among the 22 tested microorganism strains, *Piper* spices were significantly active (MIC < 0.1 mg/mL) against the anaerobes *Actinomyces israelii* and *Fusobacterium nucleatum*. The antioxidant and anti-enzymatic activities were evidenced in DPPH (10.64–82.44 mg TE/g) and ABTS (14.20–77.60 mg TE/g) radical scavenging, CUPRAC (39.94–140.52 mg TE/g), FRAP (16.05–77.00 mg TE/g), chelating (0–34.80 mg EDTAE/g), anti-acetylcholinesterase (0–2.27 mg GALAE/g), anti-butyrylcholinesterase (0.60–3.11 mg GALAE/g), anti-amylase (0.62–1.11 mmol ACAE/g) and anti-glucosidase (0–1.22 mmol ACAE/g) assays. Several *Piper* extracts (10 μg/mL) inhibited both melanin synthesis (to 32.05–60.65% of αMSH+ cells) and release (38.06–45.78% of αMSH+ cells) in αMSH-stimulated B16F10 cells, partly explained by their tyrosinase inhibitory properties. Our study uncovers differences between *Piper* spices and sheds light on their potential use as nutraceuticals or cosmeceuticals for the management of different diseases linked to bacterial infections, Alzheimer’s dementia, type 2 diabetes mellitus or hyperpigmentation.

## 1. Introduction

The genus *Piper* (Piperaceae, pepper) gathers around 1500–2000 species of herbs, shrubs, vines or trees, widely distributed in the tropical regions of southeast Asia and Oceania, Western and Central Africa, Central and South America [1,2]. Numerous members of the genus are valued as spices and have been used for thousands of years as flavor, color and aroma enhancers and preservatives [3]. Considered the ‘king of spices’ due to its massive share in the global spice market, *P. nigrum* L. is also used in traditional Chinese and Ayurvedic medicine to treat fever, colds, colic and gastrointestinal ailments [4]. Nevertheless, the fruits of *P. nigrum* (peppercorns) are differently processed, leading to numerous commercial varieties. For instance, black peppers represent the unripe berries allowed to dry until a wrinkled dark layer is formed, whereas white peppers are ripe fruits from which the outer layer has been removed. Green peppers are unripe berries picked at the same stage of ripeness as black peppercorns, while red peppers are unhulled ripe berries; both green and red peppercorns are dried under special color-preserving conditions [3].

The oblong, blunt and blackish-green fruits of *P. longum* L. are also among the most important species in the genus, being ethnomedicinally recommended as analgesic, immune-modulator, aphrodisiac or emmenagogue to treat respiratory or gastrointestinal disorders, post-partum hemorrhage, epilepsy, diabetes and rheumatoid arthritis [5,6]. Also elongated in shape, the fruits of *P. retrofractum* Vahl. (syn. *P. chaba* Hunter, *P. officinarum* (Miq.) C. DC.) are traditionally used as digestive, tonic, carminative in asthma, bronchitis, gastrointestinal ulcers, diarrhea or postpartum hemorrhage [1,7].

Spices from the genus *Piper* represent a promising lead in natural product research, as extracts or extract-derived bioactive compounds have been shown to act as multiple pharmacological agents with antioxidant, antimicrobial, anti-proliferative, anti-inflammatory, anti-hyperglycemic, anti-hyperlipidemic, cardio-vasculo-protective and neuroprotective effects [4,6]. Among the diverse specialized metabolites produced by the genus *Piper* (e.g., essential oils, flavonoids, lignans), piperamides are considered the key bioactive molecules [2]. As the main constituent of *P. nigrum* and *P. longum,* piperine was shown to possess analgesic, anti-depressant, anti-inflammatory and hepatoprotective effects in cell-free, cell-based and animal models [8]. Acyl coenzyme A-cholesterol acyltransferase, 5′-adenosine monophosphate-activated protein kinase-p38 mitogen-activated protein kinase signaling pathway, P-glycoprotein and different ion channels are among the most promising molecular targets of piperine [9,10]. In addition, other piperamides, such as pipernonaline, retrofractamide B, guineensine, *N*-isobutyl-2,4-octadecadienamide, *N*-isobutyl-2,4,14-eicosatrienamide and piperanine exhibited gastro-protective effects in indomethacin- and ethanol-induced gastric lesions in rats, whereas piperolein B, retrofractamide C, pipernonaline and dehydropipernonaline were reported to act as diacylglycerol acyltransferase inhibitors, with putative implications in the treatment of obesity [11]. Furthermore, guineensine has been shown to be a nanomolar endocannabinoid reuptake inhibitor with potent in vivo cannabimimetic effects [12,13].

As a continuation of our multi-group research efforts to screen for new phytochemical drug leads for the management of infectious diseases, Alzheimer’s disease, type 2 diabetes mellitus and skin disorders, the biological and metabolite profiling of *P. nigrum* (black, green, white and red), as well as of *P. longum* and *P. retrofractum*, was performed. The comprehensive phytochemical characterization of *Piper* spices was carried out by state-of-the-art spectro-chromatographic techniques, whereas the antibacterial and antifungal properties were assessed on a panel of reference microorganisms. The antioxidant and anti-enzymatic activities were investigated with regard to radical scavenging, metal reducing and chelating, anti-cholinesterase, anti-amylase, anti-glucosidase and anti-tyrosinase assays. Lastly, the anti-melanogenic effects were evaluated in murine melanoma B16F10 cells stimulated with α-melanocyte stimulating hormone (αMSH).

## 2. Materials and Methods

### 2.1. Plant Material and Extraction

Dried fruits (peppercorns) of various *Piper* spices (Table 1) were bought from various local European markets, identified by the authors (A.T., K.S.W.) and stored in Department of Pharmacognosy, *Grigore T. Popa* University of Medicine and Pharmacy Iasi (Romania). The dried peppercorns (20 g from each material) were pulverized with a grinding machine (Rommelsbacher ElektroHausgeräte GmbH, Dinkelsbühl, Germany) and subjected to ultrasound-assisted extraction with methanol (200 mL) for 30 min at 40 °C, for three consecutive times. The filtered and pooled extracts were evaporated to dryness. The extraction yields are provided for each sample in Table 1.

### 2.2. Phytochemical Characterization

Total phenolic content (TPC) and total flavonoid content (TFC) of *Piper* methanolic extracts were determined as previously described [14,15], with the results expressed as mg gallic acid equivalents (GAE)/g extract for TPC and mg rutin equivalents (RE)/g extract for TFC. The profiling of piperamides was performed by liquid chromatography with high resolution tandem mass spectrometry (LC-HRMS/MS), based on a method detailed by Luca et al. [2]. The quantitative estimation of piperamides was carried out by liquid chromatography with diode array detection (LC-DAD), as previously presented [2]; the data are reported as mg piperine equivalents (PE)/g extract.

### 2.3. Antimicrobial Assay

The methanolic extracts of *Piper* spices were screened for their antibacterial and antifungal activities by micro-dilution method according to the European Committee on Antimicrobial Susceptibility Testing [16]. Mueller-Hinton (MH) broth and MH broth with 5% lysed sheep blood were used for the growth of non-fastidious and fastidious bacteria, respectively, whereas MH broth with 2% glucose was used for the growth of fungi. Minimum inhibitory concentrations (MIC) of the samples were determined in a panel of reference microorganisms. The following strains were used: Gram-positive bacteria (*Staphylococcus aureus* ATCC 25923, *Staphylococcus epidermidis* ATCC 12228, *Micrococcus luteus* ATCC 10240, *Bacillus cereus* ATCC 10876, *Enterococcus faecalis* ATCC 29212, *Streptococcus mutans* ATCC 25175, *Actinomyces israelii* ATCC 10049 and *Clostridium perfringens* ATCC 13124); Gram-negative bacteria (*Campylobacter jejunii* ATCC 33291, *Salmonella* Typhimurium ATCC14028, *Escherichia coli* ATCC 25922, *Proteus mirabilis* ATCC 12453, *Klebisiella pneumoniae* ATCC 13883, *Pseudomonas aeruginosa* ATCC 9027l, *Bacteroides fragilis* ATCC 10240, *Prevotella intermedia* ATCC 25611, *Fusobacterium nucleatum* ATCC 25586 and *Veillonella parvula* ATCC 10790); fungi (*Candida glabrata* ATCC 90030, *C. albicans* ATCC 102231 and *C. parapsilosis* ATCC 22019).

### 2.4. Antioxidant, Cholinesterase, Amylase and Glucosidase Assays

The antioxidant activity of *Piper* samples was determined by 1,1-diphenyl-2-picryhydrazyl (DPPH) and 2,2′-azino-bis(3-ethylbenzothiazoline-6-sulfonic acid) (ABTS) radical scavenging, cupric reducing antioxidant capacity (CUPRAC), ferric reducing antioxidant power (FRAP), metal chelating (MCA) and phosphomolybdenum (PBD) assays [14,15]. DPPH, ABTS, CUPRAC and FRAP activities are expressed as mg trolox equivalents (TE)/g extract. In MCA, the data were presented as mg EDTA equivalents (EDTAE)/g extract, whereas in PBD assay, the results are provided are mmol TE/g extract. Acetylcholinesterase (AChE) and butyrylcholinesterase (BChE) inhibition was assessed as described in [14,15] and expressed as mg galanthamine equivalents (GALAE)/g extract, while amylase and glucosidase inhibition [14,15] is reported as mg acarbose equivalents (ACAE)/g extract.

### 2.5. Cell Viability Assay

Murine melanoma B16F10 cells (ATCC^®^ CRL-6475, LGC Standards, Łomianki, Poland) and immortalized human keratinocyte HaCaT cells (Cell Lines Service GmbH, Eppelheim, Germany) were maintained in Dulbecco’s Modified Eagle’s Medium (DMEM) containing 4.5 g/L glucose and supplemented with 10% fetal bovine serum (FBS). The cells were cultured at 37 °C in a humidified atmosphere with 5% CO_2_. Neutral red uptake (NRU) assay was performed as described previously by Repetto et al. [17]. Briefly, after seeding in 96-well plates (3 × 10^3^ cells/well), cells were grown overnight and treated with different concentrations of *Piper* methanolic extracts (10–200 μg/mL) or piperine (10–200 μg/mL). Control cells, containing appropriate volumes of the solvent (DMSO), were kept under the same conditions. After 72 h (for B16F10 cells) or 48 h (for HaCaT cells), the cells were incubated for 3 h with neutral red solution (33 µg/mL) in conditioned medium containing 1% FBS. The cell morphology was examined with an inverted microscope (Nikon Eclipse, Nikon, Japan) and documented using an Invenio II camera (DEltaPix, Smørum, Denmark). The cells were then rinsed with Dulbecco’s phosphate buffered saline (DPBS) and lysed using acidified ethanol solution (50% *v*/*v* ethanol, 1% *v*/*v* acetic acid and 49% H_2_O). The absorbance of the released neutral red was measured at λ = 540 nm using a FilterMax F5 microplate reader (Molecular Devices, San Jose, CA, USA) and corrected by the absorbance at λ = 620 nm. Mean measured values for the lysate from control cells were set as 100% and used to calculate the percentage of cell viability in the other samples.

### 2.6. Melanin Assay

After seeding in 6-well plates (0.5 × 10^5^ cells/well), B16F10 cells were grown over night and treated with *Piper* methanolic extracts (10 μg/mL), piperine (10 μg/mL) or the positive control kojic acid (10 μg/mL). Melanin production was stimulated with αMSH (10 nM). After 72 h, the conditioned medium and cell pellets were collected. Negative control cells, containing appropriate volumes of solvent (DMSO), were kept under the same conditions, but in the absence of αMSH stimulation. Next, cell pellets were dissolved in 1 N NaOH and incubated for 2 h at 80 °C. Conditioned media and cell lysates were then transferred to a 96-well plate and the absorbance was measured at λ = 405 nm using the FilterMax F5 microplate reader. The content of protein in cell lysates was established by Bradford assay [18]. The melanin released in the conditioned medium and the melanin content in cell lysates (μg melanin/mg protein) were calculated with the help of synthetic melanin calibration curves. Mean measured values obtained for αMSH-stimulated control cells without the tested samples (αMSH+) were set as 100% and used to calculate the percentage of melanin release/content in the other samples.

### 2.7. Tyrosinase Assay

#### 2.7.1. Mushroom Tyrosinase Assay

The inhibition of the monophenolase and diphenolase activities of mushroom tyrosinase was performed as previously described [19,20]. For the monophenolase inhibitory assay, 100 mM phosphate buffer (PBS, pH = 6.8, 80 μL) was mixed with 20 μL of *Piper* methanolic extracts (10 μg/mL), piperine (10 μg/mL) or kojic acid (10 μg/mL). After incubation at 25 °C for 10 min with 500 U/mL mushroom tyrosinase (20 μL), 2 mM L-tyrosine (80 μL) was added to the samples and incubated at 25 °C for additional 20 min. In the case of diphenolase inhibitory assay, PBS (120 μL) and samples (20 μL) were incubated for 10 min at 25 °C with mushroom tyrosinase (80 μL). Following the addition of 4 mM L-DOPA (40 μL), the samples were further incubated for additional 20 min at 25 °C. In both assays, the control, containing appropriate volumes of solvent (DMSO), was obtained under the same conditions. Next, the formation of dopachrome was measured spectrophotometrically at λ = 450 nm using the FilterMax F5 microplate reader. Mean measured values obtained for the solvent control were set as 100% and used to calculate the percentage of enzyme activity in the other samples.

#### 2.7.2. Murine Tyrosinase Assay

The inhibition of murine tyrosinase activity was assessed using B16F10 cell lysate, as previously described [19,20]. The B16F10 cells (8 × 10^6^) were lysed for 1 h with 1% Triton X-100 in PBS (5 mL) in an ultrasound ice-cold water bath. After centrifugation (10 min at 13,000 rpm), the supernatant was collected as murine tyrosine solution and the protein concentration was measured using a DC Protein Assay kit (Bio-Rad, Hercules, CA, USA). Cell lysate containing 20 μg protein was mixed with 20 μL of *Piper* methanolic extracts (10 μg/mL), piperine (10 μg/mL) or kojic acid (10 μg/mL), 4 mM L-DOPA (40 μL) and PBS (up to 200 μL). The control, containing appropriate volumes of solvent (DMSO), was obtained under the same conditions. After incubation for 4 h at 37 °C, the absorbance was measured spectrophotometrically at λ = 450 nm using the FilterMax F5. Mean measured values obtained for the solvent control were set as 100% and used to calculate the percentage of enzyme activity in the other samples.

### 2.8. Statistical Analysis

All non-cell-based tests were performed in three replicates, and the results are expressed as mean ± standard deviation (S.D.). The cell-based assays were performed in triplicate, with data representative for at least three individual experiments and expressed as mean ± standard error of mean (S.E.M.). Statistical analysis was performed in OriginPro2020 (OriginLab) using ANOVA with Turkey’s post hoc test; *p* < 0.05 was considered statistically significant.

## 3. Results and Discussion

### 3.1. Phytochemical Characterization

*Piper* fruits are acknowledged as valuable and rich sources of a wide range of specialized metabolites, such as piperamides, flavonoids, lignans and essential oils [6]. In the current work, the methanolic fruits extracts of *P. nigrum*, *P. longum* and *P. retrofractum* were phytochemically characterized with regard to TPC, TFC as well as the piperamides’ profile (LC-HRMS/MS) and content (LC-DAD).

TPC was generally higher in the six varieties of black pepper (36.71–58.90 mg GAE/g) than in *P. longum* (29.53 mg GAE/g) and *P. retrofractum* (32.60 mg GAE/g) (Table 1). TFC showed a high variability, with *P. retrofractum* (19.70 mg RE/g) displaying the highest values, followed by green pepper (18.37 mg RE/g); on the contrary, black pepper 3 (1.44 mg RE/g) exhibited a very low TFC (Table 1). Our results are comparable with previous literature data. For instance, Zarai et al. [21] reported 37.48 mg GAE/g and 3.01 mg/g quercetin equivalents/g in the methanol extract of *P. nigrum*.

The LC-HRMS/MS profiling of piperamides was performed according to dereplication strategies carefully presented in a previous work for a series of hexane *Piper* extracts [2]. To estimate the piperamides’ content, the chromatographic and spectrometric LC-HRMS/MS identification data (UV, HRMS, HRMS/MS, molecular formula) from Table 2 were correlated with the LC-DAD data, as detailed in [2].

Piperolactam C (**1**) and piperlongumine (**2**) were solely found in *P. retrofractum*, whereas piperyline (**3**) was present only in *P. nigrum* (5.81–15.40 mg/g). The amount of piperlonguminine (**4**) varied from 2.72 mg/g in white pepper to 16.87 mg/g in *P. longum*. Piperine (**5**) was by far the most abundant constituent in almost analyzed samples, with its highest values in white and green peppers (308.36–315.19 mg/g); in comparison, *P. retrofractum* had ~3-fold lower values of piperine. Nevertheless, the major piperamide in *P. retrofractum* was represented by pellitorine (**8**; 206.95 mg/g), a constituent found in levels 5 to 25 times lower in the other spices. Quantified as sum of stereoisomers, piperettines (**6**) showed significantly high amounts in *P. nigrum* varieties (21.51–70.60 mg/g), while their content was very low in the other two investigated *Piper* species (Table 2).

Piperolein A (**7**) and piperolein B (**14**) were absent in *P. longum*, whereas their levels in the other spices varied from 5.84 mg/g (red pepper) to 28.97 mg/g (white pepper) and from 2.70 mg/g (*P. retrofractum*) to 17.26 mg/g (black pepper 1), respectively. Pipercallosine (**9**), pipernonaline (**11**; 47.62 mg/g) and *N*-Isobutyl-2,4,12-octadecatrienamide (**17**; 82.65 mg/g) reached their highest values in *P. longum*. On the contrary, samples of *P. nigrum* were characterized by the highest amounts of dehydropipernonaline (**10**; 22.23 mg/g), neopeollitorine B (**12**; 7.89 mg/g), retrofractamide B (**13**; 22.07 mg/g), piperundecalidine (**15**; 7.66 mg/g) and guineensine (**16**; 42.53 mg/g). The total piperamide content revealed a variable profile from 247.75 to 591.42 mg/g, in the following increasing order: red pepper < *P. retrofractum* < black pepper 3 < *P. longum* < white pepper ~ black pepper 2 < green pepper~black pepper 1 (Table 3).

### 3.2. Antimicrobial Activity

The extensive usage of antibiotics and the rapid emergence of multi-drug resistant pathogens represent serious issues for modern medicine. Numerous solutions are under current investigation, including the screening and identification of novel plant-based antimicrobials with superior efficiency and safety profiles [22]. In the present study, the antimicrobial activity of the eight methanolic fruit extracts of *Piper* spp. was assessed by micro-dilution method, with results reported as MIC values (Table 4). The sensitivity of the tested strains varied based on the *Piper* variety/species, microorganism group or species. The criteria proposed by Kuete and Efferth [23] were used to categorize the observed antimicrobial activity into three groups: significant activity (MIC < 0.1 mg/mL), moderate activity (0.1 < MIC < 0.625 mg/mL) and weak/no activity (MIC > 0.625 mg/mL). For instance, a moderate activity (MIC = 0.125–0.5 mg/mL) was noticed against *S. aureus* for black peppers 2 and 3, white pepper, green pepper and *P. retrofractum*, whereas only green and red peppers were found to be active against *S. epidermidis.* Except for *P. longum*, all other investigated spices presented a moderate activity against *M. luteus*, with black peppers 1 and 2 and green pepper as the most potent agents. Black peppers 1 and 3, white pepper and *P. retrofractum* exhibited MIC values of 0.5 mg/mL against *Bacillus cereus*. The anaerobic *A. israelii* showed a particular sensitivity toward all *Piper* samples (MIC = 0.0625–0.5 mg/mL), with black pepper 3 and white pepper displaying the strongest inhibitory effects. The investigated spices also demonstrated weak activity against aerobic Gram-negative bacteria (MIC > 2 mg/mL), whereas the anaerobic Gram-negative bacteria (*B. fragilis, P. intermedia, F. nucleatum, V. parvula*) were considerably more sensitive. For instance, a MIC value of 0.0625 mg/mL was noticed for black pepper 3, green pepper and *P. retrofractum* against *F. nucleatum*, while black peppers 1–2, white and red peppers exhibited a MIC value of 0.125 mg/mL against the same pathogen. Concerning the yeast strains, all spices (except for *P. longum*) displayed moderate antifungal activity (MIC = 0.25–0.5 mg/mL), but only against *C. parapsilosis* (Table 4).

Previously, *P. nigrum* exhibited MIC values within a similar range (0.0625–0.5 mg/mL) against *S. aureus*, *S. epidermidis*, *Staphylococcus xylosus*, *B. cereus*, *E. faecalis*, *Listeria monocytogenes*, *P. aeruginosa*, *Salmonella Typhi*, *S. Typhimurium* or *E. coli* [21,24,25,26]. Regarding the antibacterial mechanisms of action, black pepper was shown to induce plasmolysis, decrease cell membrane permeability, inhibit the tricarboxylic acid pathway, increase intracellular pyruvic acid and reduce intracellular ATP levels [25]. The antibacterial properties of *P. longum* against *S. aureus*, *S. pyogenes*, *K. pneumoniae* [27] and *P. retrofractum* against *B. subtilis*, *S. aureus*, *E. faecalis*, *E. coli*, *K. pneumonia*, *P. aeruginosa*, *S. Typhi*, *Vibrio parahaemolyticus* or *C. albicans* [28] have also been investigated.

However, to the best of our knowledge, the significant antibacterial activity of *Piper* spices against the anaerobic pathogens tested in this study has not been previously reported. Due to their slow growth rate, poly microbial nature and high resistance to conventional antimicrobial chemotherapy, the management of anaerobic infections is very challenging. *Actinomyces* spp. are often responsible for head and neck infections, aspiration pneumonia, intracranial abscesses or chronic mastoiditis, whereas *Bacteroides* spp. are predominant in intra-abdominal and other infections that originate from the gut flora. *Prevotella* spp. have been isolated from respiratory infections and their complications, while *Veillonella* spp. have been involved in periodontitis, osteomyelitis or endocarditis [29,30].

### 3.3. Antioxidant Activity

Used for more than 50 years, synthetic antioxidants, such as *tert*-butylhydroquinone, propylgallate or butylated hydroxytoluene, are nowadays incriminated for carcinogenicity and banned in numerous countries [31,32]. Consequently, there is a high demand, especially from the food industry, for novel natural antioxidants as food preservatives. Previously, different *Piper* extracts have been shown to possess promising antioxidant effects, as evaluated by DPPH radical, superoxide radical, hydrogen peroxide, nitric oxide scavenging, FRAP, MCA, PBD or β-carotene bleaching assays [21,33,34,35,36,37,38,39].

**Figure 1:**
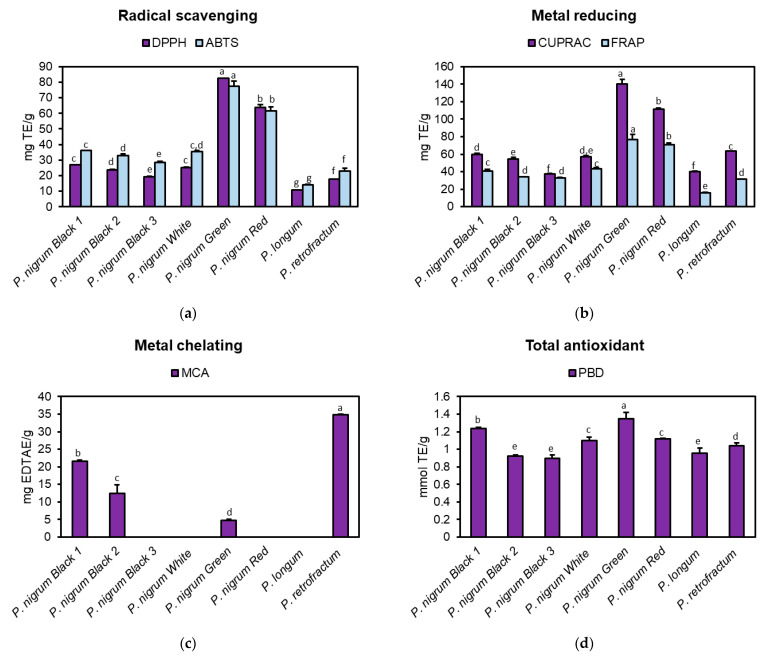
Antioxidant activity of the methanolic fruit extracts obtained from different spices of genus *Piper* in (**a**) radical scavenging, (**b**) metal reducing, (**c**) metal chelating and (**d**) total antioxidant assays. Data are presented as mg Trolox equivalents (TE)/g extract in 1,1-diphenyl-2-picrylhydrazyl (DPPH), 2,2′-azino-bis(3-ethylbenzothiazoline) 6-sulfonic acid (ABTS) radical scavenging, cupric reducing antioxidant capacity (CUPRAC) and ferric reducing antioxidant power (FRAP) assays, mg EDTA equivalents (EDTAE)/g extract in metal chelating assay (MCA) and mmol TE/g extract in phosphomolybdenum (PBD) assay. Each bar represents mean ± S.D. of three determinations; samples sharing different superscript letters are significantly different at *p* < 0.05.

In the current study, the antioxidant activity of the eight methanolic fruits extracts of *Piper* spp. was investigated through radical scavenging, metal chelating, metal reducing and PBD assays. Green and red peppers exhibited the most potent DPPH and ABTS radical scavenging activities (82.44 and 63.67 mg TE/g in DPPH assay for green and red peppers, respectively; 77.60 and 61.63 mg TE/g in ABTS assay for green and red peppers, respectively) (Figure 1a). The scavenging activity of the other samples decreased in the following order: black pepper 1 > white pepper > black pepper 2 > black pepper 3 > *P. retrofractum* > *P. longum*. A similar trend was observed in metal reducing assays (Figure 1b). CUPRAC varied from 37.36 mg TE/g in black pepper 3 to 140.52 mg/g TE in green pepper, whereas FRAP varied from 16.05 mg TE/g in *P. longum* and 77.00 mg/g TE in green pepper. In MCA (Figure 1c), only black pepper 1 (21.54 mg EDTAE/g), green pepper (4.64 mg EDTAE/g), black pepper 2 (12.35 mg EDTAE/g) and *P. retrofractum* (34.80 mg EDTAE/g) were found to be active. PBD assay (Figure 1d) revealed good total antioxidant properties of all investigated spices, with green pepper (1.35 mmol TE/g) and black pepper 1 (1.24 mmol TE/g) as the most potent spices.

With respect to the phytochemical composition (Table 1), it can be noticed that the most active samples in DPPH, ABTS, FRAP, CUPRAC and PBD assays were those with the highest TPC, whereas the four active samples in MCA were represented by those with the highest TFC; this could suggest the involvement of these two groups of specialized metabolites in the observed antioxidant properties.

### 3.4. Anti-Cholinesterase Activity

Alzheimer’s disease (AD) is the most common cause of dementia and mainly affects the elderly population, provoking progressive cognitive decline, memory impairment and psycho-behavioral disorders. The presence of senile plaques, neurofibrillary tangles as well as a decrease in the cholinergic transmission are often noticed in the brain of patients with AD [40,41]. The inhibition of AChE and BChE, two sister enzymes that are responsible for the cleavage of the neurotransmitter acetylcholine, is one of the main currently investigated therapeutic strategies, with numerous natural products displaying promising anti-cholinesterase activity [42].

**Figure 2:**
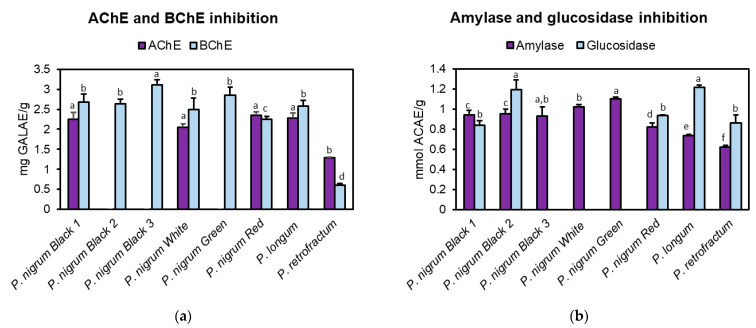
Acetylcholinesterase, butyrylcholinesterase, glucosidase and amylase inhibitory activity of the methanolic fruit extracts obtained from different spices of genus *Piper* in (**a**) cholinesterase and (**b**) amylase and glucosidase assays. Data are presented as mg galanthamine equivalents (GALAE)/g extract in acetylcholinesterase (AChE) and butyrylcholinesterase (BChE) assays and mg acarbose equivalents (ACAE)/g extract in amylase and glucosidase assays. Each bar represents mean ± S.D. of three determinations; samples sharing different superscript letters are significantly different at *p* < 0.05.

Within the frame of this study, all *Piper* spices exhibited moderate anti-BChE effects, with values ranging from 0.60 mg GALAE/g (in *P. retrofractum*) to 3.11 mg GALAE/g (in black pepper 3). In contrast, slightly lower anti-AChE properties were noticed, especially considering that, three spices (black peppers 2 and 3 and green pepper) were inactive, with highest activity observed at 2.35 mg GALAE/g in red pepper (Figure 2a). The anti-cholinesterase properties of the genus *Piper* have scarcely been reported. For instance, the ethyl acetate extract of *P. nigrum* exhibited efficient inhibition percentages of 61.5% against AChE and 90.6% against BChE at 100 μg/mL, whereas from the 21 isolated piperamides, piperine was the most potent inhibitor with IC_50_ values of 63.16 μg/mL in AChE and 25.11 μg/mL in BChE, respectively [43]. On the other hand, the dichloromethane extract of *P. longum*, as well as piperine (IC_50_ = 0.32 mM), pipercide (IC_50_ = 0.61 mM) and guineensine (IC_50_ = 1.61 mM), were shown to be able to inhibit AChE [44]. In another study, the methanolic extracts obtained from the fruits of *P. nigrum* and *P. retrofractum* displayed IC_50_ values of 11.13 and 14.08 μg/mL against AChE, respectively [45].

### 3.5. Anti-Amylase and Anti-Glucosidase Activity

Type 2 diabetes mellitus is a chronic metabolic disorder characterized by high blood glucose levels as a consequence of an inefficient insulin functionality or secretion [46]. Inhibiting amylase and glucosidase, two key pancreatic enzymes that convert dietary polysaccharides into absorbable monosaccharides, represents a promising therapeutic approach. Several synthetic inhibitors (acarbose, voglibose, miglitol) are currently available, but they are often reported to produce serious side effects (flatulence, abdominal pain, diarrhea, and hepatotoxicity) [47]. Plants have been shown to be inexhaustible reservoirs of bioactive molecules with potential inhibitory activity against the two enzymes targeted in the management of type 2 diabetes [48]. As presented in Figure 2b, it can be noticed that black pepper 3, white pepper and green pepper were ineffective against glucosidase, whereas the activity of the remaining spices varied from 0.84 mmol ACAE/g (black pepper 1) to 1.22 mmol ACAE/g (*P. longum*). The extracts displayed anti-amylase effects in the following order: *P. retrofractum* < *P. longum* < red pepper < black peppers 1–3 < white pepper < green pepper. Previously, the ethanolic extract of *P. nigrum* and methanolic extract of *P. longum* were reported to possess anti-glucosidase activities, with IC_50_ values of 216 and 112.90 μg/mL, respectively [47,49]. In addition, various piperamides isolated from *P. nigrum*, *P. longum* or *P. retrofractum*, such as piperine, pipercyclobutanamide D, piperoside, pellitorine, brachystamide B, guineensine, and pipataline, were shown to exert significant to moderate glucosidase and, sometimes, amylase inhibitory effects [46,49,50].

### 3.6. Anti-Melanogenic Activity

Synthetized by the melanocytes and then transferred to keratinocytes, melanin is the main pigment in hair, skin and eyes. Nevertheless, the excessive production and accumulation of melanin can lead to hyperpigmentation disorders, such as melasma, senile lentigo or freckles [51,52]. Modulation of the melanogenesis by skin-lightening agents represents a promising therapeutic approach. However, the available drugs, such as kojic acid, arbutin, or hydroquinone, are banned in numerous countries, due to their serious side effects, such as irreversible depigmentation, dermatitis or skin cancer [52,53]. Therefore, there is an urgent need to screen for new safer depigmentation agents.

**Figure 3:**
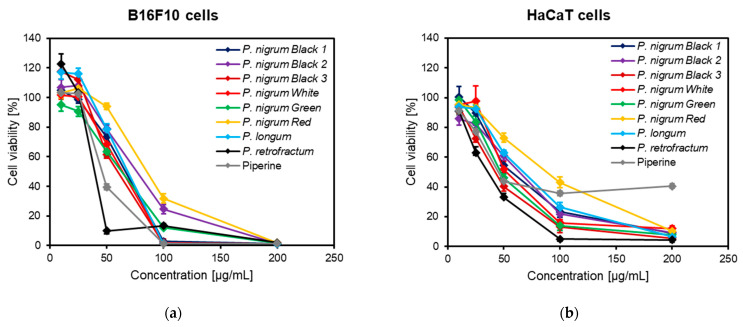
Concentration–response curves (10–200 μg/mL) showing the effect of the methanolic fruit extracts obtained from different spices of genus *Piper* and piperine in (**a**) B16F10 and (**b**) HaCaT cells. Cells were seeded in 96-well plates (3 × 10^3^/well) and incubated for 72 h (B16F10 cells) or 48 h (HaCaT cells) with different concentrations of *Piper* extracts (10–200 μg/mL) or piperine (10–200 μg/mL). Data are presented as percentage cell viability (%) in comparison to the solvent (DMSO)-treated control cells, set at 100%.

The potential melanin inhibitory effects of spices from the genus *Piper* were investigated in αMSH-stimulated murine melanoma B16F10 cells. Prior to the melanin assay, the influence of the *Piper* extracts on the viability of B16F10 cells and immortalized human HaCaT keratinocytes was investigated by the neutral red uptake assay. Despite a pronounced cytotoxicity, especially at higher concentrations (100–200 μg/mL), no significant changes in either B16F10 or HaCaT cell viability was noticed at 10 μg/mL (Figure 3); therefore, this concentration was considered safe and selected as test concentration for the melanin assay.

**Figure 4:**
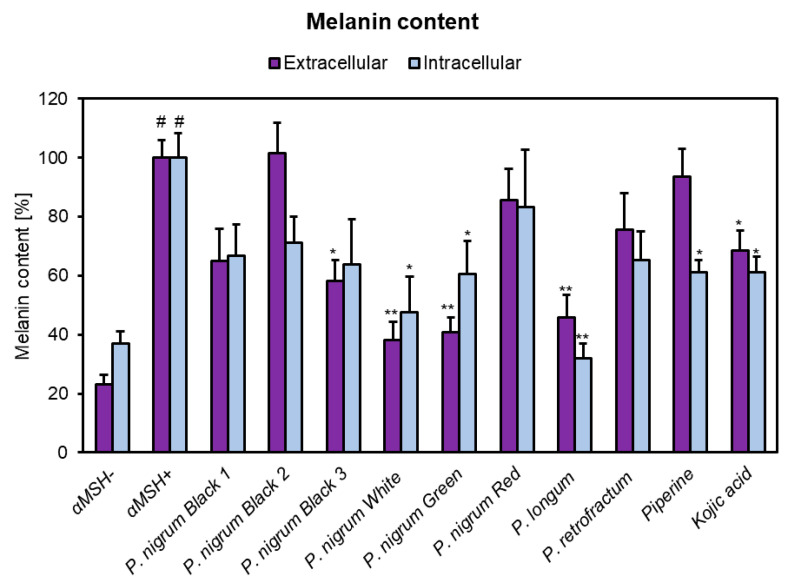
Melanin inhibitory activity in αMSH-stimulated B16F10 cells of the methanolic fruit extracts obtained from different spices of genus *Piper*, piperine and kojic acid. B16F10 cells were seeded in 6-well plates (0.5 × 10^5^/well) and incubated for 72 h with *Piper* extracts (10 μg/mL), piperine (10 μg/mL) or kojic acid (10 μg/mL); melanin production was stimulated with αMSH (10 nM). Data are presented as the percentage of intracellular and extracellular melanin content in comparison to αMSH stimulated control (αMSH+) cells, set at 100%. Each bar represents the mean ± S.E.M. of at least three independent experiments performed in triplicates; # *p* <0.001 vs. non-stimulated (αMSH-) cells; * *p* < 0.05; ** *p* < 0.01; vs. αMSH+ cells.

As compared to the non-treated cells (αMSH–), the extracellular melanin content (=melanin release in the conditioned medium) and intracellular melanin content (=melanin content in the lysate) were both significantly increased by 2.7- and 4.3-fold in αMSH+ cells, respectively (Figure 4). When αMSH+ cells were treated with *Piper* samples (10 μg/mL), a significant reduction of intracellular melanin content was noticed in white pepper (47.72% of αMSH+ cells), green pepper (60.65% of αMSH+ cells) and, especially, *P. longum* (32.05% αMSH+ cells). Furthermore, the same extracts also decreased the melanin release, with a relative extracellular melanin content of 38.06%, 40.68% and 45.78% of αMSH+ cells, respectively. Piperine (10 μg/mL) was only able to reduce the intracellular melanin content (61.01% of αMSH+ cells), whereas the extracellular melanin release was not considerably impaired. Kojic acid (10 μg/mL), the positive control for the melanin assay, significantly downregulated both the intracellular (61.13% of αMSH+ cells) and extracellular (68.59% of αMSH+ cells) melanin content.

To the best of our knowledge, there are no previous studies reporting on the anti-melanogenic activity of extracts obtained from various *Piper* spices. Nevertheless, the potential of different piperamides to inhibit the melanin production in αMSH-stimulated B16F10 cells has been documented. For instance, piperlongumine significantly reduced the total melanin production, by reducing the activity of tyrosinase and expression of several pro-melanogenic proteins, such as tyrosinase-related protein-1 (TRP-1), TRP2, microphthalmia-associated transcription factor (MITF) [54]. On the other hand, piperlonguminine decreased the production of melanin by downregulating tyrosine expression at the transcription level via a reduction of cyclic adenosine monophosphate responsive element binding protein (CREB) phosphorylation [55,56].

### 3.7. Anti-Tyrosinase Activity

**Figure 5:**
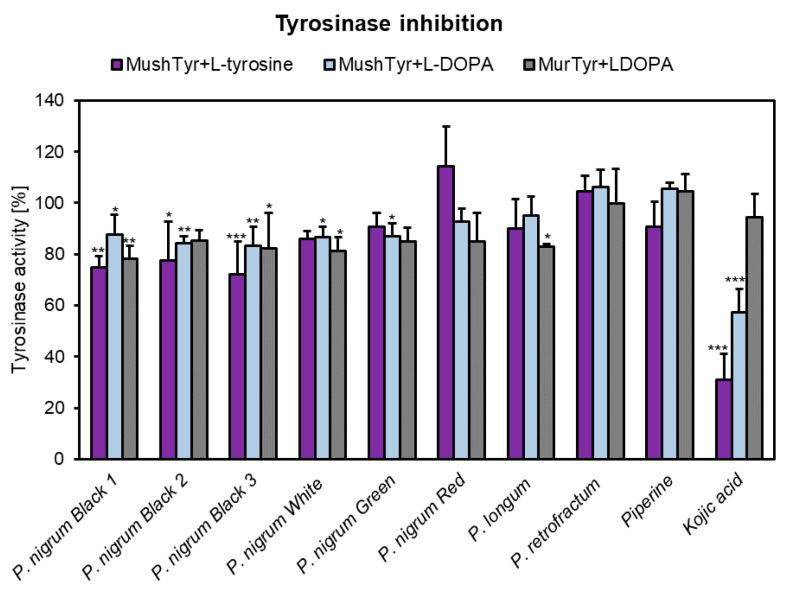
Tyrosinase inhibitory activity of the methanolic fruit extracts (10 μg/mL) obtained from different spices of genus *Piper*, piperine (10 μg/mL) and kojic acid (10 μg/mL). Data are presented as percentage of tyrosinase activity in comparison to solvent (DMSO) control, set at 100%. Each bar represents the mean ± S.D. of three determinations; * *p* < 0.05; ** *p* < 0.01; *** *p* < 0.001 vs. control.

To investigate whether the anti-melanogenic effects of *Piper* spices in αMSH-stimulated B16F10 cells might be exerted via tyrosinase inhibition, monophenolase and diphenolase assays were performed. Tyrosinase is a key enzyme of melanogenesis, catalyzing the rate-limiting conversion of L-tyrosine to L-dihydroxyphenylalanine (L-DOPA) and subsequently to dopaquinone. Numerous plant extracts have previously been shown to exert tyrosinase inhibitory effects [20,57,58,59]. The influence on the monophenolase activity was investigated with mushroom tyrosinase (by adding L-tyrosine as substrate), whereas the influence on the diphenolase activity was evaluated with both mushroom and murine tyrosinase (by adding L-DOPA as substrate) (Figure 5). It was noticed that only the three black pepper samples (10 μg/mL) significantly reduced the mushroom monophenolase activity (by 22–27%). With respect to the mushroom diphenolase, black, green and white peppers showed moderate anti-tyrosinase effects (reductions by 13–16%). On the other hand, the activity of murine tyrosinase was significantly impaired by black (1–2) and white peppers and, additionally, by *P. longum* (activities between 78.36–85.22%). Piperine was unable to modulate the tyrosinase activity, whereas kojic acid, the positive control, decreased the mushroom monophenolase and diphenolase activity to 30.86% and 57.27%, respectively, with no significant effects on murine tyrosinase. Previously, the anti-tyrosinase activity of various *Piper* species, such as *P. retrofractum, P. caninum* Blume, *P. abbreviatum* Opiz, *P. erecticaule* C.DC., *P. stylosum* Miq., *P. magnibaccum* C.DC., and *P. betle* L., has been reported [60,61,62,63].

It is worth mentioning that the decrease in the melanin content in B16F10 cells treated with *Piper* extracts might result not only from the direct modulation of tyrosinase activity, but also from the transcriptional control of genes involved in the melanogenesis pathway or post-transcriptional modification of tyrosinase leading to enzyme degradation [64]. The potential involvement of *Piper* compounds in these pathways requires further investigation.

## 4. Conclusions

In this work, the phytochemical and multifunctional biological profiling of four varieties of *P. nigrum* (black, green, white and red) and two other *Piper* species, namely *P. longum* and *P. retrofractum*, was presented. Firstly, a comprehensive metabolite characterization by TPC, TFC, LC-HRMS/MS and LC-DAD was performed, revealing significant levels of bioactive piperamides, such as piperine, piperlongumine, piperlonguminine, retrofractamide B, guineensine, pipernonaline, or *N*-isobutyl-2,4,12-octadecatrienamide. Then, the spices were assessed for their antibacterial and antifungal properties on a panel of 22 reference microorganisms. Beside a moderate activity against *Staphylococcus aureus*, *S. epidermidis, M. luteus*, *B. cereus* and *C. parapsilosis* (MIC = 0.125–0.5 mg/mL), several samples, such as black, green, white peppers or *P. retrofractum*, were particularly active (MIC = 0.0625–0.125 mg/mL) against anaerobe species, such as *A. israelii, B. fragilis, P. intermedia, F. nucleatum* and *V. parvula*. A potent antioxidant activity of the investigated spices was revealed, as evaluated by several complementary tests, such as radical scavenging, metal reducing, chelating and phosphomolybdenum assays. With respect to the modulation of enzymes targeted in the management of Alzheimer’s disease (acetylcholinesterase, butyrylcholinesterase) and type 2 diabetes mellitus (amylase, glucosidase), the current data suggest moderate inhibitory effects of *Piper* spices against the investigated enzymes. Several *Piper* extracts, namely white pepper, green pepper and *P. longum*, inhibited both the melanin synthesis (to 32.05–60.65% of α-MSH+ cells) and melanin release (38.06–45.78% of α-MSH+ cells) in α-MSH-stimulated B16F10 cells at 10 μg/mL, a non-toxic concentration in both B16F10 and HaCaT cells. The anti-melanogenic properties could be partly explained by the moderate inhibition of murine tyrosinase (tyrosinase activities between 78.36–85.22%).

By evaluating the poly pharmacological potential of a series of *Piper* varieties and species, globally available and used as spices, our study shows similarities and differences between the investigated *Piper* samples. This could inspire future research into the use of *Piper* nutraceuticals or cosmeceuticals in the management of infectious diseases, Alzheimer’s dementia, type 2 diabetes mellitus or hyperpigmentation disorders.

## Figures and Tables

**Table 1:** Extraction yields, total phenolic and flavonoid contents in the methanolic fruit extracts obtained from different spices of genus *Piper*.

Sample		Voucher	Extraction Yield	TPC (mg GAE/g)	TFC (mg RE/g)
*P. nigrum* L.	Black1	PN1/102019	10.5%	40.96 ± 0.42 ^b^	10.03 ± 0.22 ^b^
	Black2	PN5/102019	10.9%	40.80 ± 0.45 ^b^	9.12 ± 0.32 ^b^
	Black3	PN6/102019	11.9%	36.71 ± 0.18 ^c^	1.44 ± 0.15 ^e^
	White	PN2/1020219	7.7%	42.11 ± 1.06 ^b^	4.66 ± 0.30 ^c^
	Green	PN3/102019	12.5%	58.90 ± 1.84 ^a^	18.37 ± 0.27 ^a^
	Red	PN4/102019	14.5%	41.41 ± 1.00 ^b^	5.42 ± 0.32 ^c^
*P. longum* L.		PL1/112019	18.3%	29.53 ± 0.25 ^d^	3.01 ± 0.03 ^d^
*P. retrofractum* Vahl.		PR1/112019	10.1%	32.60 ± 0.22 ^e^	19.70 ± 0.13 ^d^

Data are presented as mg gallic acid equivalents (GAE)/g extract for the total phenolic content (TPC) and mg rutin equivalents (RE)/g extract for total flavonoid content (TFC) and represent the mean values ± S.D. of three determinations; values in the same column sharing different superscript letters are significantly different at *p* < 0.05.

**Table 2:** LC-HRMS/MS data of piperamides tentatively identified the methanolic fruit extracts obtained from different spices of genus *Piper*.

No.	Compound	UV [nm]	HRMS (+) [*m*/*z*]	MF	HRMS/MS (+) [*m*/*z*]
**1**	Piperolactam C	220; 340	310.1075	C_18_H_15_NO_4_	193.0750; 120.0395
**2**	Piperlongumine	230; 330	318.1360	C_17_H_19_NO_5_	269.0957; 221.0805; 201.0527
**3**	Piperyline	255; 310; 342	272.1291	C_16_H_17_NO_3_	201.0555; 173.0572; 143.0503; 135.0405; 115.0571
**4**	Piperlonguminine	230; 340	274.1452	C_16_H_19_NO_3_	161.0576; 135.0452
**5**	Piperine	240; 310; 341	286.1443	C_17_H_19_NO_3_	201.0542; 173.0525; 135.0455; 112.0733
**6**	Piperettines	310; 345	312.1605	C_19_H_21_NO_3_	227.0679; 199.0776; 169.0555; 141.085; 131.0405
**7**	Piperolein A	260; 340	316.1904	C_19_H_25_NO_3_	231.1056; 161.0538; 135.0512
**8**	Pellitorine	210; 260	224.2015	C_14_H_25_NO	168.1414; 151.1170; 123.1196; 109.0603
**9**	Pipercallosine	210; 265; 310	330.2049	C_20_H_27_NO_3_	259.1312; 241.1301; 208.1725; 135.0490
**10**	Dehydropipernonaline	210; 265	340.1919	C_21_H_25_NO_3_	227.1001; 161.0554; 131.0414
**11**	Pipernonaline	215; 260; 305	342.2065	C_21_H_27_NO_3_	229.1564; 199.0916; 161.0593; 135.0471
**12**	Neopeollitorine B	210; 265	236.2001	C_15_H_25_NO	151.1140; 109.0625
**13**	Retrofractamide B	210; 260	356.2203	C_22_H_29_NO_3_	283.1309; 255.1371; 234.1822; 215.1868; 135.0433
**14**	Piperolein B	210; 265; 305	344.2205	C_21_H_29_NO_3_	259.1299; 222.1829; 135.0395
**15**	Piperundecalidine	215; 270	368.2223	C_23_H_29_NO_3_	246.1807; 215.1046; 173.0559; 135.0411
**16**	Guineensine	215; 260; 315	384.2586	C_24_H_33_NO_3_	311.1985; 283.2033; 161.0754; 135.0577
**17**	*N*-Isobutyl-2,4,12-octadecatrienamide	220; 260	334.3099	C_22_H_39_NO	261.2253; 233.2201

HRMS, high-resolution mass spectra; MF, molecular formula; T_R_ retention time; UV, ultraviolet.

**Table 3:** Piperamide content in the methanolic fruit extracts obtained from different spices of genus *Piper*.

No.	Compound	*P. nigrum*						*P. longum*	*P. retrofractum*
		Black 1	Black 2	Black 3	White	Green	Red		
		mg PE/g Extract							
**1**	Piperolactam C	nq	nq	nq	nq	nq	nq	nq	18.17 ± 0.25 ^a^
**2**	Piperlongumine	nq	nq	nq	nq	nq	nq	nq	14.19 ± 0.14 ^a^
**3**	Piperyline	11.66 ± 0.17 ^c^	12.68 ± 0.16 ^b^	8.62 ± 0.18 ^e^	10.90 ± 0.08 ^d^	15.40 ± 0.34 ^a^	5.81 ± 0.07 ^f^	nq	nq
**4**	Piperlonguminine	4.90 ± 0.12 ^d^	4.84 ± 0.26 ^d^	2.83 ± 0.13 ^e^	2.72 ± 0.15 ^f^	6.01 ± 0.14 ^c^	5.10 ± 0.13 ^d^	16.87 ± 0.13 ^a^	12.93 ± 0.03 ^b^
**5**	Piperine	276.70 ± 0.82 ^d^	299.63 ± 0.99 ^c^	293.07 ± 0.71 ^c^	308.36 ± 0.50 ^b^	315.19 ± 1.12 ^a^	154.03 ± 0.21 ^f^	249.79 ± 0.99 ^e^	93.86 ± 0.13 ^g^
**6**	Piperettines	70.60 ± 1.69 ^a^	64.65 ± 0.46 ^b^	46.12 ± 0.67 ^c^	66.18 ± 0.87 ^b^	70.69 ± 0.06 ^a^	21.51 ± 0.08 ^d^	3.64 ± 0.40 ^e^	nq
**7**	Piperolein A	28.83 ± 1.60 ^a^	17.96 ± 0.28 ^c^	9.93 ± 0.16 ^d^	28.97 ± 0.66 ^a^	23.90 ± 0.54 ^b^	5.84 ± 0.12 ^e^	nq	23.00 ± 1.21 ^b^
**8**	Pellitorine	39.68 ± 0.14 ^b^	15.95 ± 0.10 ^e^	22.41 ± 0.26 ^d^	26.11 ± 0.17 ^c^	18.78 ± 0.15 ^e^	9.33 ± 0.07 ^f^	7.90 ± 0.35 ^g^	206.95 ± 0.22 ^a^
**9**	Pipercallosine	3.72 ± 0.51 ^c^	3.28 ± 0.15 ^d^	3.02 ± 0.06 ^d^	4.72± 0.37 ^b^	4.57 ± 0.18 ^b^	2.04 ± 0.04 ^e^	9.25 ± 0.47 ^a^	nq
**10**	Dehydropipernonaline	22.23 ± 0.29 ^a^	11.18 ± 0.09 ^e^	18.50 ± 0.46 ^b^	17.12 ± 0.17 ^c^	12.77 ± 0.12 ^d^	2.68 ± 0.04 ^f^	12.71 ± 0.29 ^d^	nq
**11**	Pipernonaline	3.37 ± 0.12 ^c^	nq	2.14 ± 0.20 ^d^	5.60 ± 0.44 ^b^	nq	nq	47.62 ± 0.29 ^a^	nq
**12**	Neopeollitorine B	7.89 ± 0.65 ^a^	3.26 ± 0.10 ^c^	2.73 ± 0.20 ^d^	2.31 ± 0.36 ^d^	3.14 ± 0.01 ^c^	nq	nq	6.51 ± 0.25 ^b^
**13**	Retrofractamide B	22.07 ± 0.57 ^a^	13.28 ± 0.21 ^d^	6.14 ± 0.06 ^f^	6.75 ± 0.31 ^e^	19.75 ± 0.21 ^b^	12.65 ± 0.12 ^d^	14.63 ± 0.07 ^c^	nq
**14**	Piperolein B	17.26 ± 0.10 ^a^	12.46 ± 0.27 ^d^	11.02 ± 0.01 ^e^	13.17 ± 0.24 ^c^	15.35 ± 0.20 ^b^	3.20 ± 0.06 ^f^	nq	2.70 ± 0.01 ^g^
**15**	Piperundecalidine	7.66 ± 0.46 ^a^	4.07 ± 0.09 ^c^	4.86 ± 0.06 ^b^	nq	4.28 ± 0.16 ^c^	4.19 ± 0.10 ^c^	6.32 ± 0.19 ^d^	7.48 ± 0.11 ^a^
**16**	Guineensine	42.53 ± 0.08 ^a^	14.18 ± 0.33 ^e^	28.57 ± 0.12 ^c^	11.95 ± 0.04 ^f^	29.59 ± 0.16 ^b^	10.22 ± 0.05 ^g^	24.51 ± 0.25 ^d^	29.73 ± 0.05 ^b^
**17**	*N*-Isobutyl-2,4,12-octadecatrienamide	32.31 ± 0.37 ^d^	14.18 ± 0.21 ^e^	49.44 ± 0.33 ^b^	13.61 ± 0.19 ^f^	37.62 ± 0.20 ^c^	12.64 ± 0.05 ^g^	82.65 ± 0.25 ^a^	1.98 ± 0.05 ^h^
	*Total piperamides*	591.42 ± 4.01 ^a^	525.35 ± 4.10 ^b^	588.42 ± 6.44 ^a^	247.75 ± 0.87 ^f^	530.67 ± 1.49 ^b^	453.58 ± 7.63 ^d^	475.89 ± 1.05 ^c^	423.72 ± 2.49 ^e^

Data are presented as mg piperine equivalent (PE)/g extract and represent the mean values ± S.D. of three determinations; values in the same row sharing different superscript letters are significantly different at *p* < 0.05.

**Table 4:** Antimicrobial activity of the methanolic fruit extracts obtained from different spices of genus *Piper*.

Microbial Species	*P. nigrum*						*P. longum*	*P. retrofractum*	*Reference drug*
	Black 1	Black 2	Black 3	White	Green	Red			
	MIC (mg/mL)								MIC (mg/L)
*S. aureus* ATCC 25923	4	0.25	0.5	0.125	0.5	1	4	0.25	0.98 ^1^
*S. epidermidis* ATCC 12228	>4	2	4	4	0.125	0.5	4	4	0.98 ^1^
*M. luteus* ATCC 10240	0.125	0.125	0.25	0.125	0.25	0.5	1	0.5	0.12 ^1^
*B. cereus* ATCC 10876	0.5	1	0.5	0.5	2	2	4	0.5	0.98 ^1^
*E. faecalis* ATCC 29212	>4	>4	>4	4	4	>4	4	>4	1.95 ^1^
*S. mutans* ATCC 25175	4	4	4	4	4	4	>4	4	0.98 ^1^
*A. israelii* ATCC 10049	0.125	0.125	0.0625	0.0625	0.125	0.5	0.5	0.25	0.5 ^1^
*C. perfringens* ATCC 13124	2	2	2	2	4	4	4	2	1.95 ^3^
*C. jejunii* ATCC 33291	4	>4	2	4	4	>4	>4	>4	0.125 ^2^
*S.* Typhimurium ATCC14028	>4	>4	>4	>4	4	>4	>4	>4	0.061 ^2^
*E. coli* ATCC 25922	>4	>4	>4	>4	4	>4	>4	>4	0.015 ^2^
*P. mirabilis* ATCC 12453	>4	>4	>4	>4	2	2	>4	>4	0.03 ^2^
*K. pneumoniae* ATCC 13883	>4	>4	>4	>4	4	>4	>4	>4	0.122 ^2^
*P. aeruginosa* ATCC 9027l	>4	>4	>4	>4	4	>4	>4	>4	0.488 ^2^
*B. fragilis* ATCC 10240	0.5	0.25	0.25	0.25	0.5	1	1	1	0.98 ^3^
*P. intermedia* ATCC 25611	0.25	0.25	0.25	0.25	0.25	>4	0.5	0.25	0.488 ^3^
*F. nucleatum* ATCC 25586	0.125	0.125	0.0625	0.125	0.0625	0.125	0.25	0.0625	1.95 ^3^
*V. parvula* ATCC 10790	0.25	0.25	0.125	0.25	0.25	1	1	2	1.95 ^3^
*C glabrata* ATCC 90030	2	2	2	4	2	4	4	2	0.24 ^4^
*C. albicans* ATCC 102231	1	1	0.5	1	1	1	2	1	0.48 ^4^
*C. parapsilosis* ATCC 22019	0.5	0.5	0.25	0.5	0.5	0.5	2	0.5	0.24 ^4^

Minimum inhibitory concentrations (MIC) present representative values of three determinations; the superscript numbers indicate the use of (^1^) vancomycin, (^2^) ciprofloxacin, (^3^) metronidazole and (^4^) nystatin as the standard drugs towards Gram-positive bacteria, Gram-negative bacteria, anaerobes and yeasts, respectively.

## Data Availability

Data is contained within the article.

## References

[1] Mgbeahuruike E.E., Yrjönen T., Vuorela H., Holm Y. (2017). Bioactive compounds from medicinal plants: Focus on *Piper* species. S. Afr. J. Bot..

[2] Luca S.V., Minceva M., Gertsch J., Skalicka-Woźniak K. (2021). LC-HRMS/MS-based phytochemical profiling of Piper spices: Global association of piperamides with endocannabinoid system modulation. Food Res. Int..

[3] Takooree H., Aumeeruddy M.Z., Rengasamy K.R., Venugopala K.N., Jeewon R., Zengin G., Mahomoodally M.F. (2019). A systematic review on black pepper (*Piper nigrum* L.): From folk uses to pharmacological applications. Crit. Rev. Food Sci. Nutr..

[4] Salehi B., Zakaria Z.A., Gyawali R., Ibrahim S.A., Rajkovic J., Shinwari Z.K., Khan T., Sharifi-Rad J., Ozleyen A., Turkdonmez E. (2019). *Piper* species: A comprehensive review on their phytochemistry, biological activities and applications. Molecules.

[5] Kumar S., Kamboj J., Sharma S. (2011). Overview for various aspects of the health benefits of *Piper longum* Linn. fruit. J. Acupunct. Meridian Stud..

[6] Yadav V., Krishnan A., Vohora D. (2020). A systematic review on *Piper longum* L.: Bridging traditional knowledge and pharmacological evidence for future translational research. J. Ethnopharmacol..

[7] Islam M.T., Hasan J., Snigdha H.S.H., Ali E.S., Sharifi-Rad J., Martorell M., Mubarak M.S. (2020). Chemical profile, traditional uses, and biological activities of *Piper chaba* Hunter: A review. J. Ethnopharmacol..

[8] Shityakov S., Bigdelian E., Hussein A.A., Hussain M.B., Tripathi Y.C., Khan M.U., Shariati M.A. (2019). Phytochemical and pharmacological attributes of piperine: A bioactive ingredient of black pepper. Eur. J. Med. Chem..

[9] Kim N., Nam M., Kang M.S., Lee J.O., Lee Y.W., Hwang G.-S., Kim H.S. (2017). Piperine regulates UCP1 through the AMPK pathway by generating intracellular lactate production in muscle cells. Sci. Rep..

[10] Matsuda D., Ohte S., Ohshiro T., Jiang W., Rudel L., Hong B., Si S., Tomoda H. (2008). Molecular target of piperine in the inhibition of lipid droplet accumulation in macrophages. Biol. Pharm. Bull..

[11] Lee S.W., Rho M.-C., Park H.R., Choi J.-H., Kang J.Y., Lee J.W., Kim K., Lee H.S., Kim Y.K. (2006). Inhibition of diacylglycerol acyltransferase by alkamides isolated from the fruits of *Piper longum* and *Piper nigrum*. J. Agric. Food Chem..

[12] Reynoso-Moreno I.s., Najar-Guerrero I., Escarenño N., Flores-Soto M.E., Gertsch J.r., Viveros-Paredes J.M. (2017). An endocannabinoid uptake inhibitor from black pepper exerts pronounced anti-inflammatory effects in mice. J. Agric. Food Chem..

[13] Nicolussi S., Viveros-Paredes J.M., Gachet M.S., Rau M., Flores-Soto M.E., Blunder M., Gertsch J. (2014). Guineensine is a novel inhibitor of endocannabinoid uptake showing cannabimimetic behavioral effects in BALB/c mice. Pharmacol. Res..

[14] Uysal S., Zengin G., Locatelli M., Bahadori M.B., Mocan A., Bellagamba G., De Luca E., Mollica A., Aktumsek A. (2017). Cytotoxic and enzyme inhibitory potential of two *Potentilla* species (*P. speciosa* L. and *P. reptans* Willd.) and their chemical composition. Front. Pharmacol..

[15] Grochowski D.M., Uysal S., Aktumsek A., Granica S., Zengin G., Ceylan R., Locatelli M., Tomczyk M. (2017). In vitro enzyme inhibitory properties, antioxidant activities, and phytochemical profile of Potentilla thuringiaca. Phytochem. Lett..

[16] European Committee for Antimicrobial Susceptibility Testing (EUCAST) of the European Society of Clinical Microbiology and Infectious Diseases (ESCMID) (2003). Determination of minimum inhibitory concentrations (MICs) of antibacterial agents by broth dilution. Clin. Microbiol. Infect..

[17] Repetto G., Del Peso A., Zurita J.L. (2008). Neutral red uptake assay for the estimation of cell viability/cytotoxicity. Nat. Protoc..

[18] Bradford M.M. (1976). A rapid and sensitive method for the quantitation of microgram quantities of protein utilizing the principle of protein-dye binding. Anal. Biochem..

[19] Uchida R., Ishikawa S., Tomoda H. (2014). Inhibition of tyrosinase activity and melanine pigmentation by 2-hydroxytyrosol. Acta Pharm. Sin. B.

[20] Sabitov A., Gaweł-Bęben K., Sakipova Z., Strzępek-Gomółka M., Hoian U., Satbayeva E., Głowniak K., Ludwiczuk A. (2021). *Rosa platyacantha* Schrenk from Kazakhstan—Natural source of bioactive compounds with cosmetic significance. Molecules.

[21] Zarai Z., Boujelbene E., Salem N.B., Gargouri Y., Sayari A. (2013). Antioxidant and antimicrobial activities of various solvent extracts, piperine and piperic acid from Piper nigrum. Lwt-Food Sci. Technol..

[22] Trifan A., Luca S.V., Greige-Gerges H., Miron A., Gille E., Aprotosoaie A.C. (2020). Recent advances in tackling microbial multidrug resistance with essential oils: Combinatorial and nano-based strategies. Crit. Rev. Microbiol..

[23] Efferth T., Kuete V. (2010). Cameroonian medicinal plants: Pharmacology and derived natural products. Front. Pharmacol..

[24] Karsha P.V., Lakshmi O.B. (2010). Antibacterial activity of black pepper (*Piper nigrum* Linn.) with special reference to its mode of action on bacteria. Indian J. Nat. Prod. Res..

[25] Zou L., Hu Y.-Y., Chen W.-X. (2015). Antibacterial mechanism and activities of black pepper chloroform extract. J. Food Sci. Technol..

[26] Tang H., Chen W., Dou Z.-M., Chen R., Hu Y., Chen W., Chen H. (2017). Antimicrobial effect of black pepper petroleum ether extract for the morphology of *Listeria monocytogenes* and *Salmonella typhimurium*. J. Food Sci. Technol.

[27] Saraf A., Saraf A. (2014). Phytochemical and antimicrobial studies of medicinal plant *Piper longum* Linn. Int. J. Pharmacogn. Phytochem. Res..

[28] Panphut W., Budsabun T., Sangsuriya P. (2020). In vitro antimicrobial activity of piper retrofractum fruit extracts against microbial pathogens causing infections in human and animals. Int. J. Microbiol..

[29] Brook I. (2016). Spectrum and treatment of anaerobic infections. J. Infect. Chemother..

[30] Mitsui T., Saito M., Harasawa R. (2018). Salivary nitrate-nitrite conversion capacity after nitrate ingestion and incidence of *Veillonella* spp. in elderly individuals. J. Oral Sci..

[31] Aprotosoaie A.C., Miron A., Ciocârlan N., Brebu M., Roşu C.M., Trifan A., Vochiţa G., Gherghel D., Luca S.V., Niţă A. (2019). Essential oils of Moldavian *Thymus* species: Chemical composition, antioxidant, anti-Aspergillus and antigenotoxic activities. Flav. Fragr. J..

[32] Trifan A., Zengin G., Sinan K.I., Wolfram E., Skalicka-Woźniak K., Luca S.V. (2021). LC-HRMS/MS phytochemical profiling of *Symphytum officinale* L. and *Anchusa ochroleuca* M. Bieb.(Boraginaceae): Unveiling their multi-biological potential via an integrated approach. J. Pharm. Biomed. Anal..

[33] Gülçin İ. (2005). The antioxidant and radical scavenging activities of black pepper (*Piper nigrum*) seeds. Int. J. Food Sci. Nutr..

[34] Agbor G.A., Vinson J.A., Oben J.E., Ngogang J.Y. (2008). In vitro antioxidant activity of three *Piper* species. J. Herb. Pharmacother..

[35] Chatterjee S., Niaz Z., Gautam S., Adhikari S., Variyar P.S., Sharma A. (2007). Antioxidant activity of some phenolic constituents from green pepper (*Piper nigrum* L.) and fresh nutmeg mace (*Myristica fragrans*). Food Chem..

[36] Samudram P., Vasuki R., Rajeshwari H., Geetha A., Moorthi P.S. (2009). Antioxidant and antihepatotoxic activities of ethanolic crude extract of *Melia azedarach* and *Piper longum*. J. Med. Plants Res..

[37] Barua C., Singh A., Sen S., Barua A., Barua I. (2014). In vitro antioxidant and antimycobacterial activity of seeds of Piper longum Linn: A comparative study. SAJ Pharm. Pharmacol..

[38] Jadid N., Hidayati D., Hartanti S.R., Arraniry B.A., Rachman R.Y., Wikanta W. (2017). Antioxidant activities of different solvent extracts of *Piper retrofractum* Vahl. using DPPH assay. Proc. AIP Conf..

[39] Mahaldar K., Hossain A., Islam F., Islam S., Islam M.A., Shahriar M., Rahman M.M. (2020). Antioxidant and hepatoprotective activity of *Piper retrofractum* against paracetamol-induced hepatotoxicity in Sprague-Dawley rat. Nat. Prod. Res..

[40] Dung H.V., Cuong T.D., Chinh N.M., Quyen D., Kim J.A., Byeon J.S., Woo M.H., Choi J.S., Min B.S. (2015). Compounds from the aerial parts of *Piper bavinum* and their anti-cholinesterase activity. Arch. Pharm. Res..

[41] Ferreres F., Oliveira A.P., Gil-Izquierdo A., Valentão P., Andrade P.B. (2014). *Piper betle* leaves: Profiling phenolic compounds by HPLC/DAD–ESI/MSn and anti-cholinesterase activity. Phytochem. Anal..

[42] Gök H.N., Luca S.V., Ay S.T., Komsta Ł., Salmas R.E., Orhan I.E., Skalicka-Woźniak K. (2021). Profiling the annual change of the neurobiological and antioxidant effects of five *Origanum* species in correlation with their phytochemical composition. Food Chem..

[43] Tu Y., Zhong Y., Du H., Luo W., Wen Y., Li Q., Zhu C., Li Y. (2016). Anticholinesterases and antioxidant alkamides from Piper nigrum fruits. Nat. Prod. Res..

[44] Khatami Z., Herdlinger S., Sarkhail P., Zehl M., Kaehlig H., Schuster D., Adhami H.-R. (2020). Isolation and characterization of acetylcholinesterase inhibitors from *Piper longum* and binding mode predictions. Planta Med..

[45] Tappayuthpijarn P., Sattaponpan C., Sakpakdeecharoen I., Ittharat A. (2012). Cholinesterase inhibitory and antioxidant activities of Thai traditional remedies potentially used for Alzheimer’s disease. Thai J. East Asian Stud..

[46] Luyen B.T.T., Tai B.H., Thao N.P., Yang S.Y., Cuong N.M., Kwon Y.I., Jang H.D., Kim Y.H. (2014). A new phenylpropanoid and an alkylglycoside from *Piper retrofractum* leaves with their antioxidant and α-glucosidase inhibitory activity. Bioorg. Med. Chem. Lett..

[47] Magaña-Barajas E., Buitimea-Cantúa G.V., Hernández-Morales A., Torres-Pelayo V.d.R., Vázquez-Martínez J., Buitimea-Cantúa N.E. (2021). In vitro α-amylase and α-glucosidase enzyme inhibition and antioxidant activity by capsaicin and piperine from *Capsicum chinense* and *Piper nigrum* fruits. J. Environ. Sci. Health B.

[48] Gevrenova R., Zengin G., Sinan K.I., Yıldıztugay E., Zheleva-Dimitrova D., Picot-Allain C., Mahomoodally M.F., Imran M., Dall’Acqua S. (2021). UHPLC-MS Characterization and biological insights of different solvent extracts of two *Achillea* species (*A. aleppica and A. santolinoides*) from Turkey. Antioxidants.

[49] Pullela S.V., Tiwari A.K., Vanka U.S., Vummenthula A., Tatipaka H.B., Dasari K.R., Khan I.A., Janaswamy M.R. (2006). HPLC assisted chemobiological standardization of α-glucosidase-I enzyme inhibitory constituents from *Piper longum* Linn-An Indian medicinal plant. J. Ethnopharmacol..

[50] Huu D.M.N., Dang P.H., Huynh N.V., Dang H.P., Vuong L., Nguyen T.L.T. (2020). Pipercyclobutanamide D, a new member of the cyclobutanamide-type alkaloid, from the roots of *Piper nigrum*. J. Asian Nat. Prod. Res..

[51] Srisayam M., Weerapreeyakul N., Kanokmedhakul K. (2017). Inhibition of two stages of melanin synthesis by sesamol, sesamin and sesamolin. Asian Pac. J. Trop. Biomed..

[52] Ullah S., Park C., Ikram M., Kang D., Lee S., Yang J., Park Y., Yoon S., Chun P., Moon H.R. (2019). Tyrosinase inhibition and anti-melanin generation effect of cinnamamide analogues. Bioorg. Chem..

[53] Mustapha N., Bzéouich I.M., Ghedira K., Hennebelle T., Chekir-Ghedira L. (2015). Compounds isolated from the aerial part of Crataegus azarolus inhibit growth of B16F10 melanoma cells and exert a potent inhibition of the melanin synthesis. Biomed. Pharmacother..

[54] Jeon H.-J., Kim K., Kim Y.-D., Lee S.-E. (2019). Antimelanogenic activities of piperlongumine derived from *Piper longum* on murine B16F10 melanoma cells in vitro and zebrafish embryos *in vivo*: Its molecular mode of depigmenting action. App. Biol. Chem..

[55] Min K.R., Kim K.-S., Ro J.S., Lee S.H., Kim J.A., Son J.K., Kim Y. (2004). Piperlonguminine from *Piper longum* with inhibitory effects on alpha-melanocyte-stimulating hormone-induced melanogenesis in melanoma B16 cells. Planta Med..

[56] Kim K.S., Kim J.A., Eom S.Y., Lee S.H., Min K.R., Kim Y. (2006). Inhibitory effect of piperlonguminine on melanin production in melanoma B16 cell line by downregulation of tyrosinase expression. Pigment Cell Res..

[57] Strzępek-Gomółka M., Gaweł-Bęben K., Angelis A., Antosiewicz B., Sakipova Z., Kozhanova K., Głowniak K., Kukula-Koch W. (2021). Identification of mushroom and murine tyrosinase inhibitors from *Achillea biebersteinii* Afan. extract. Molecules.

[58] Gaweł-Bęben K., Strzępek-Gomółka M., Czop M., Sakipova Z., Głowniak K., Kukula-Koch W. (2020). *Achillea millefolium* L. and *Achillea biebersteinii* Afan. hydroglycolic extracts–bioactive ingredients for cosmetic use. Molecules.

[59] Gaweł-Bęben K., Kukula-Koch W., Hoian U., Czop M., Strzępek-Gomółka M., Antosiewicz B. (2020). Characterization of *Cistus* × *incanus* L. and *Cistus ladanifer* L. extracts as potential multifunctional antioxidant ingredients for skin protecting cosmetics. Antioxidants.

[60] Salleh W.M.N.H.W., Hashim N.A., Ahmad F., Yen K.H. (2014). Anticholinesterase and antityrosinase activities of ten *Piper* species from Malaysia. Adv. Pharm. Bull..

[61] Salleh W., Ahmad F., Khong H. (2014). Antioxidant and anti-tyrosinase activities from *Piper officinarum* C. DC (Piperaceae). J. Appl. Pharm. Sci..

[62] Hashim N.A., Ahmad F., Salleh W.M.N.H.W., Khamis S. (2019). Phytochemicals and tyrosinase inhibitory activity from *Piper caninum* and *Piper magnibaccum*. Pharm. Sci..

[63] Tan Y.P., Chan E.W.C. (2014). Antioxidant, antityrosinase and antibacterial properties of fresh and processed leaves of *Anacardium occidentale* and *Piper betle*. Food Biosci..

[64] Qian W., Liu W., Zhu D., Cao Y., Tang A., Gong G., Su H. (2020). Natural skin-whitening compounds for the treatment of melanogenesis (Review). Exp. Ther. Med..

